# Regime Shifts in a Phage-Bacterium Ecosystem and Strategies for Its Control

**DOI:** 10.1128/mSystems.00470-19

**Published:** 2019-11-05

**Authors:** Sergei Maslov, Kim Sneppen

**Affiliations:** a Department of Bioengineering, University of Illinois at Urbana-Champaign, Urbana, Illinois, USA; b Carl R. Woese Institute for Genomic Biology, University of Illinois at Urbana-Champaign, Urbana, Illinois, USA; c Center for Models of Life, Niels Bohr Institute, University of Copenhagen, Copenhagen, Denmark; University of Delhi

**Keywords:** bacteriophage therapy, bacteriophages, computer modeling, microbial communities, microbial ecology

## Abstract

The competition between bacteria often involves both nutrients and phage predators and may give rise to abrupt regime shifts between the alternative stable states characterized by different species compositions. While such transitions have been previously studied in the context of competition for nutrients, the case of phage-induced bistability between competing bacterial species has not been considered yet. Here we demonstrate a possibility of regime shifts in well-mixed phage-bacterium ecosystems. In one of the bistable states, the fast-growing bacteria competitively exclude the slow-growing ones by depleting their common nutrient. Conversely, in the second state, the slow-growing bacteria with a large burst size generate such a large phage population that the other species cannot survive. This type of bistability can be realized as the competition between a strain of bacteria protected from phage by abortive infection and another strain with partial resistance to phage. It is often desirable to reliably control the state of microbial ecosystems, yet bistability significantly complicates this task. We discuss successes and limitations of one control strategy in which one adds short pulses to populations of individual species. Our study proposes a new type of phage therapy, where introduction of the phage is supplemented by the addition of a partially resistant host bacteria.

**IMPORTANCE** Phage-microbe communities play an important role in human health as well as natural and industrial environments. Here we show that these communities can assume several alternative species compositions separated by abrupt regime shifts. Our model predicts these regime shifts in the competition between bacterial strains protected by two different phage defense mechanisms: abortive infection/CRISPR and partial resistance. The history dependence caused by regime shifts greatly complicates the task of manipulation and control of a community. We propose and study a successful control strategy via short population pulses aimed at inducing the desired regime shifts. In particular, we predict that a fast-growing pathogen could be eliminated by a combination of its phage and a slower-growing susceptible host.

## Abstract (precis)

Phage-microbe communities play an important role in human health as well as natural and industrial environments. Here we show that these communities can assume several alternative species compositions separated by abrupt regime shifts. Our model predicts these regime shifts in the competition between bacterial strains protected by two different phage defense mechanisms: abortive infection/CRISPR and partial resistance. The history dependence caused by regime shifts greatly complicates the task of manipulation and control of a community. We propose and study a successful control strategy via short population pulses aimed at inducing the desired regime shifts. In particular, we predict that a fast-growing pathogen could be eliminated by a combination of its phage and a slower-growing susceptible host.

## INTRODUCTION

Diverse ecosystems are known to be capable of regime shifts in which they abruptly and irreversibly switch between two mutually exclusive stable states (1). Such regime shifts have been extensively studied in both macroscopic and microbial ecosystems (1) and shown to be hysteretic and history dependent. In microbial ecosystems (2), these transitions are known to be possible when a bacterial species directly produces some metabolic waste products or antibiotics (3) that inhibit the growth of other bacteria. They may also occur when bacterial species compete for several food sources, which they use either in different stoichiometric ratios (4) or in different preferential orders (5). Here we explore a new type of regime shifts caused by interactions between bacteria and phages. Bacteriophages have long been known to increase bacterial diversity, especially in aquatic environments (6, 7). However, their potential to create multiple stable states with distinct bacterial species compositions so far has not been recognized. Here we illustrate a possibility of such alternative stable states and regime shifts using a computational model in which two bacterial species compete for the same food source and are simultaneously exposed to an infection by the same virulent phage. Such dual constraints are known to abate the usual competitive exclusion (8) by allowing multiple bacterial species consuming the same nutrient to coexist (7, 9).

Microbial communities are an important part of our natural and artificial surroundings and are also responsible for many aspects of human health. Some compositions of microbial communities may be useful for us, while other might be detrimental or even lethal. Thus, we would like to reliably manipulate and control the species compositions of these systems. Here we explore several strategies with the aim of controlling the state of phage-bacterium ecosystems via short population pulses inducing the desired regime shift.

## RESULTS

### Model.

We study a model describing the dynamics of two microbial species with populations *B*_1_ and *B*_2_ growing on a single limiting nutrient (e.g., carbon source) with concentration *C* and infected by a single phage species with population *P*. All populations are assumed to be well-mixed populations in an environment constantly supplied with the limiting nutrient at a rate ϕ. The dynamics of this ecosystem is given by

(1) $$\frac{\mathrm{dC}}{\mathrm{dt}}=\phi -C\delta_{C}-C\left(\frac{\lambda_{1}}{Y_{1}}B_{1}+\frac{\lambda_{2}}{Y_{2}}B_{2}\right)$$

(2) $$\frac{dB_{1}}{\mathrm{dt}}=B_{1}\left(\lambda_{1}C-\eta_{1}P-\delta_{B}\right)$$

(3) $$\frac{dB_{2}}{\mathrm{dt}}=B_{2}\left(\lambda_{2}C-\eta_{2}P-\delta_{B}\right)$$

(4) $$\frac{\mathrm{dP}}{\mathrm{dt}}=P\left(\beta_{1}\eta_{1}B_{1}+\beta_{2}\eta_{2}B_{2}-\delta_{P}\right)$$

The growth rate of each bacterial species is assumed to be proportional to the nutrient concentration *C* with species *B*_1_ growing faster than species *B*_2_: λ_1_ > λ_2_. Nutrient yields of these two species, species *B*_1_ and species *B*_2_, are given by *Y*_1_ and *Y*_2_, respectively. The phage adsorption coefficients of the two species are given by *η*_1_ and *η*_2_ and their burst sizes are *β*_1_ and *β*_2_. The two bacterial species in our model are assumed to have the same death rate *δ_B_* that also includes possible contribution from dilution of their shared environment. The death/dilution rate of the phage is given by *δ_P_*, and the nutrient is diluted at a rate *δ_C_*.

### Conditions for bistability and regime shifts.

In what follows, we explore the steady-state solutions of equations 1 to 4, the only asymptotic dynamical behavior possible in our system. In the absence of phages, the faster growing species *B*_1_ would always eliminate the slower growing species *B*_2_ due to competitive exclusion (8). Phages in principle allow for a slow-growing species to coexist with the fast-growing one or even to completely take over the ecosystem. In order for this to happen in high-nutrient/high-phage environments, species *B*_2_ needs to be less susceptible to phage infections than species *B*_1_: *λ*_1_/*η*_1_ < *λ*_2_/*η*_2_. In the extreme case, where species *B*_2_ is fully resistant to the phage (*η*_2_ = 0), the coexistence between these bacterial species has been previously identified and computationally studied (7, 9, 10).

Here we introduce and study another regime of a phage-bacterium ecosystem in which two bacterial species could mutually exclude each other. This falls under the category of discontinuous and abrupt regime shifts between alternative stable states in microbial ecosystems (see reference 2 for a review), which have been previously modelled in the context of competition for nutrients (4, 5) and without phages. In order for a phage-bacterium ecosystem to be in principle capable of bistability, the slow-growing bacterial species needs to produce disproportionately more phages per each unit of consumed nutrient than the fast-growing one: *Y*_2_*β*_2_ > *Y*_1_*β*_1_. As we show in the supplemental material, the bistability requires the following three inequalities to be satisfied:

(5) $$\lambda_{1}>\lambda_{2}$$

(6) $$\frac{\lambda_{1}}{\eta_{1}}<\frac{\lambda_{2}}{\eta_{2}}$$

(7) $$\frac{\lambda_{1}}{Y_{1}\beta_{1}\eta_{1}}>\frac{\lambda_{2}}{Y_{2}\beta_{2}\eta_{2}}$$

**FIG 1:**
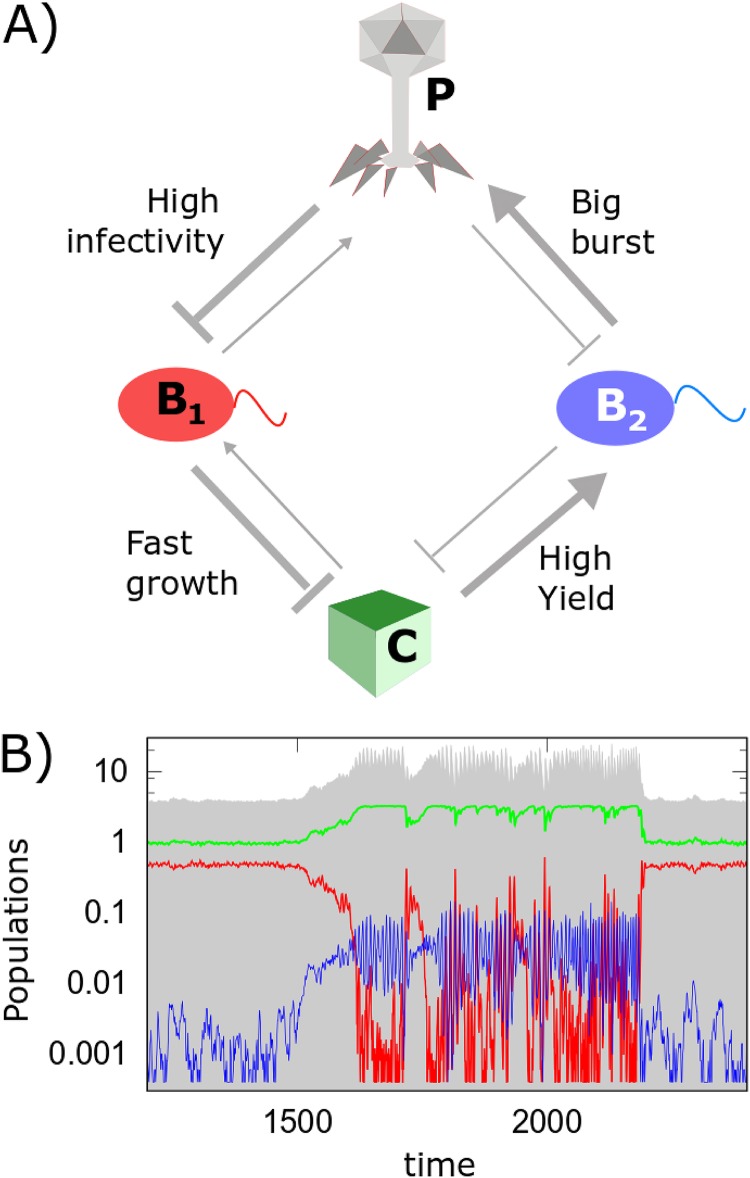
Alternative stable states and regime shifts in a phage-bacterium ecosystem. (A) Diagram of interactions between the three species and one nutrient resource in our model: the fast-growing (red *B*_1_) and the slow-growing (blue *B*_2_) bacterial species are limited by the same nutrient *C* and infected by the same phage *P*. The slow-growing bacteria are more protected from infections by phage, but if infected, they generate a larger burst size. The negative effective interaction from *B*_1_ to *B*_2_ is mediated via the nutrient, while that from *B*_2_ to *B*_1_ is mediated via the phage. (B) Representative stochastic simulation of the model. Note the abrupt and large regime shifts of the ecosystem between two alternative stable states dominated by bacteria *B*_1_ and *B*_2_. All populations are always maintained above a very low level (4 × 10^–4^) provided by a weak influx of species to the ecosystem. Both phage and nutrient concentrations experience a discontinuous shift up if the ecosystem suddenly flips from the *B*_1_-dominated state to the *B*_2_-dominated one and down in the opposite case. The model parameters are λ_1_ = 1, λ_2_ = 0.8, *Y*_1_ = *Y*_2_ = 1, η_1_ = 0.20, η_2_ = 0.15, β_1_ = 2, β_2_ = 40, δ*_C_* = δ*_B_* = δ*_P_*, and *ϕ* = 0.66.

Figure 1A illustrates the basic mechanisms responsible for bistability and regime shifts in our ecosystem. The thickness of each arrow scales with the relative strength of the interaction between the nodes it connects. Thus, the width of the arrow pointing from the nutrient to the bacterial species *B_i_* reflects its growth rate λ*_i_*, while the width of the arrow pointing in the opposite direction represents the rate *λ*_*i*_/*Y*_*i*_ at which this bacterial species depletes the nutrient. Similarly, the width of the arrow pointing from the phage to the bacterial species *B_i_* reflects its adsorption coefficient *η_i_*, while that of the arrow going in the opposite direction—the rate *β_i_η_i_* at which this bacterial species generates new phages.

Figure 1B shows a stochastic simulation of our model with parameters *λ*_1_ = 1, *λ*_2_ = 0.8, *Y*_1_ = *Y*_2_ = 1, η_1_ = 0.20, η_2_ = 0.15, β_1_ = 2, β_2_ = 40, δ*_C_* = δ*_B_* = δ*_P_* = 0.2, and ϕ = 0.66 (see Materials and Methods for details). In our simulations, we do not allow the population of either of three species (*B*_1_, *B*_2_, and *P*) to fall below a very small value 4 × 10^– 4^. This is equivalent to keeping a constant but weak influx of these species to the ecosystem. As a result, each species would start growing as soon as the ecosystem’s internal parameters would make its net growth rate positive.

Random fluctuations in population sizes of bacteria and phages could trigger spontaneous regime shifts between two alternative stable states of the ecosystem visible in Fig. 1B. One of these states is dominated by the fast-growing bacterial species *B*_1_. It suppresses the slow-growing species *B*_2_ by the virtue of competitive exclusion via their shared nutrient. In the second stable state, the slow-growing species *B*_2_ with a large burst size *β*_2_, generates such a high population of phages that they completely eliminate the fast-growing species *B*_1_, which is relatively more susceptible to phage infections. This steady state also has a larger nutrient concentration due to a lower rate of its depletion by species *B*_2_.

### History dependence of the ecosystem state.

**FIG 2:**
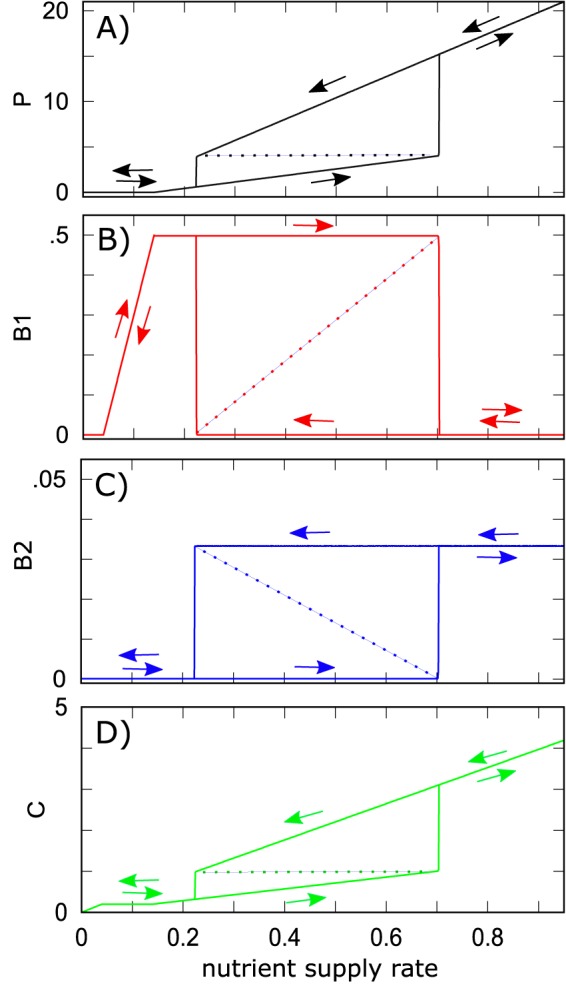
Hysteresis loops in populations of phage *P* (black) (A), fast-growing bacteria *B*_1_ (red) (B), slow-growing bacteria *B*_2_ (blue) (C), and nutrient concentration *C* (green) (D) as the nutrient supply rate ϕ (*x* axis) is changed first up from 0 to 1 and then down to 0. Note two sudden discontinuous transitions (regime shifts) at both ends of the hysteresis loop. The dashed lines mark populations in the dynamically unstable state separating two alternative stable states. Parameters of the model are the same as in Fig. 1, except for a varying nutrient supply rate *ϕ* (*x* axis) and the absence of stochastic fluctuations.

When equations 5 to 7 are satisfied, the bistability is possible only in a certain intermediate range of the nutrient supply rate. Figure 2A to D shows the changes in steady-state values of *P*, *B*_1_, *B*_2_, and *C*, respectively, when the nutrient supply rate *ϕ* is slowly changed first up from 0 to 1 and then down to 0 again. For very low nutrient supply rates *ϕ* <0.04, neither bacteria nor phages can survive, and the system stays abiotic *B*_1_ = *B*_2_ = *P* = 0. The fast-growing bacteria *B*_1_ first appears for *ϕ* ≥ 0.04 and prevents the appearance of the slow-growing species due to competitive exclusion. As the nutrient supply rate is increased above 0.14, the population of the phage *P* becomes sustainable and linearly increases with *ϕ*. *B*_2_ continues to be competitively excluded until much higher rate of nutrient supply *ϕ*^(1)^ = 0.70, at which the ecosystem undergoes a regime shift to the state dominated by *B*_2_ and excluding *B*_1_. This alternative stable state persists all the way up to the nutrient supply rate. The growth of *B*_1_ is prevented by a high phage population to which this species is especially susceptible. When *ϕ* is lowered, the *B*_2_-dominated state survives down to the nutrient supply rate *ϕ*^(2)^ = 0.23, which is much lower than *ϕ*^(1)^ = 0.70. Thus, for nutrient supply rates between 0.23 and 0.70, the ecosystem is bistable and can be in any of the two alternative stable states making upper and lower parts of the hysteresis loops in Fig. 2A to D. Note that the population of phages and the concentration nutrients generally change in synchrony: when *B*_1_ is dominant, both phage and nutrient levels are low, while the dominance of *B*_2_ generates many phages which significantly lower its population and prevent it from fully exploiting resources, thereby keeping *C* high.

### Controlling regime shifts by population pulses.

Phages have recently been investigated as potential agents of control of populations of individual bacterial species in the gut microbiome (11). However, when alternative stable states are present, the state of an ecosystem is complicated by hysteresis and history dependence.

One may need to switch a microbial ecosystem from an undesirable/diseased state to a desirable/healthy state without perturbing the environmental parameters such as nutrient supply rate. One way to achieve such control is by adding a fixed amount of one of species *P*, *B*_1_, *B*_2_, or of the nutrient *C* giving rise to an instantaneous increase of its current population/concentration. Such one-time addition, which we call a “population pulse,” is similar to the “impulsive control strategy” discussed in reference 12. Since *P*, *C*, and *B*_2_ are all higher in the *B*_2_-dominated state than in the *B*_1_-dominated state, adding a population pulse of either one of them to the *B*_1_-dominated state could, in principle, trigger a regime shift. Similarly, adding a population pulse of *B*_1_ to the *B*_2_-dominated state could result in a regime shift in the opposite direction.

**FIG 3:**
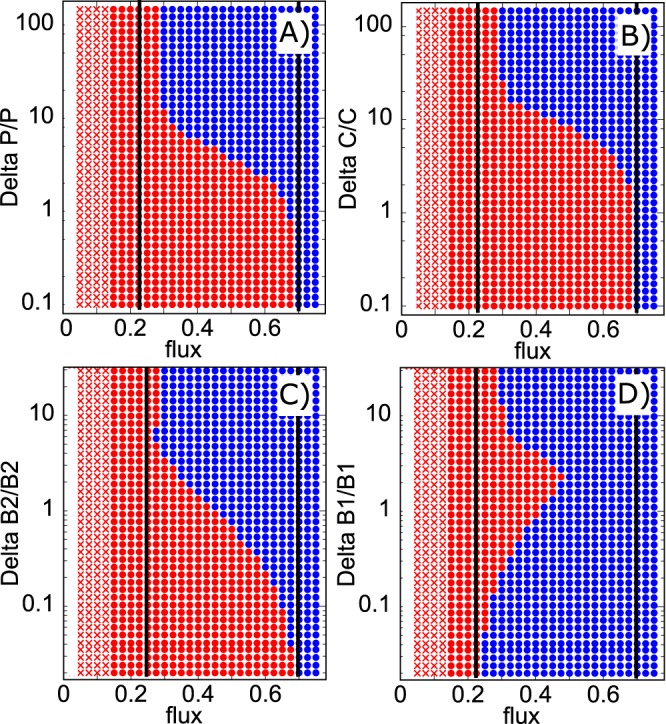
Control of the ecosystem by a pulse in phage population *P* (A), resource concentration *C* (B), bacterial populations *B*_2_ (C) or *B*_1_ (D). Red symbols mark the *B*_1_-dominated state, while blue symbols mark the *B*_2_-dominated state. In the region marked with red crosses, phages cannot exist: *P* = 0. The *x* axis is the nutrient supply rate *ϕ* with the bistable region confined between two black solid lines. The *y* axis is the magnitude of the pulse normalized by the population/concentration of the target stable state, that is to say, by that of the *B*_2_-dominated state in panels A to C and of the *B*_1_-dominated state in panel D. For nutrient supply rates 0.27 < ϕ < 0.7, the *B*_1_-dominated state (red) can be switched to the *B*_2_-dominated state (red) by adding a sufficiently large pulse of phage *P* (A), nutrient *C* (B), or bacteria *B*_2_ (C). Conversely, for 0.23 < ϕ < 0.46, the *B*_2_-dominated state (blue) can be switched to the *B*_1_-dominated state (red) by adding a sufficiently large pulse of bacteria *B*_1_ (D).

Figure 3 explores successes and limitations of the population pulse strategy. We found that this strategy works but only within a certain range of nutrient supply that is generally more narrow than the bistability region itself. A regime shift from the *B*_1_- to the *B*_2_-dominated state can be triggered across the entire bistability region. Conversely, a regime shift from the *B*_2_- to the *B*_1_-dominated state by adding a pulse of *B*_1_ can be made only for *ϕ* below 0.46, which is lower than *ϕ*^(1)^ = 0.7 − the upper bound of the bistable region (the right solid black line in Fig. 3). Another observation is the reentrant transition in Fig. 3D; adding too much of *B*_1_ to the *B*_2_-dominated state may prevent the regime shift from taking place. We also note that in order to trigger a regime shift one generally needs to add a pulse that would transiently make the population of the perturbed species to exceed its steady-state value in the targeted state (pulse normalized to 1 on the *y* axis in Fig. 3). Indeed, a pulse changes only one out of four populations/concentrations in our ecosystem. Thus, it needs to be large enough to drive the remaining three populations in the general direction of the regime shift.

Consider a situation where we can simultaneously perturb all three species and the nutrient and set their populations/concentrations (*C*, *B*_1_, *B*_2_, and *P*) to any desired value. In this case, transient populations after a pulse could be made smaller than their steady-state values in the target state. Indeed, to switch the state of the ecosystem, it would be sufficient to make all four populations/concentrations just a little bit closer to the target state than their values in the dynamically unstable state shown as dashed lines in Fig. 2A to D.

### Model with perfect abortive infection in *B*_1_.

In one of the phage defense mechanisms called abortive infection (Abi) (13), phages enter and kill the host without producing any phage progeny. A special limit of our model is obtained when species *B*_1_ is characterized by abortive infection: *β*_1_ = 0, while *η*_1_>0. Our equations in this case predict *ϕ*^(1)^ = *∞*, which means that *B*_1_ would not disappear from the ecosystem for any nutrient supply *ϕ*. Indeed, this species generates no phage progeny; thus, it can always outcompete a small amount of the slower-growing species *B*_2_ infected by phages. However, analogous to Fig. 3A and C, a sufficiently large population pulse of *B*_2_ and *P* can become established in the system and eliminate *B*_1_. This could happen for *ϕ*>*ϕ*^(2)^.

## DISCUSSION

We introduced a mathematical model of regime shifts in phage-bacterium ecosystems. The alternative stable states in our model are populated by different bacterial species mutually excluding each other. The negative interactions between these species are mediated by either their coinfecting phages or their shared nutrients. In this respect, the mechanism of bistability in our model is similar to that in consumer resource models without phages (4). Indeed, the mandatory (but not sufficient) condition for bistability in either of these two models is a significant difference in stoichiometry of competing microbial species. In our model, this stoichiometry is quantified by *Y*β—the product of nutrient yield and burst size of a given bacterial species. It can be interpreted as the conversion factor connecting the amount of nutrients used to build a single bacterial cell to the number of phages it produced upon lysis. Comparison of inequalities in equation 6 and equation 7 shows that bistability is possible only when conversion factors of two bacterial species are sufficiently different from each other: *Y*_2_*β*_2_ > *Y*_1_*β*_1_.

Similarly, multistability studied in references 4 and 14 requires species competing for two types of essential resources (e.g., C and N) to have different C/N stoichiometries.

Regime shifts and multistability are known to occur when competition between species in principle allows for their coexistence, while the differences in stoichiometry make such coexistence dynamically unstable (4, 14). This is also true in our model, where bistability between species *B*_1_ and *B*_2_ is possible whenever their coexistence is dynamically unstable. Conversely, a dynamically stable coexistence of *B*_1_ and *B*_2_ is possible whenever inequalities given by equations 5 and 6 are satisfied, while that in equation 7 changes the direction to $\lambda_{1}/(Y_{1}\beta_{1}\eta_{1})<\lambda_{2}/(Y_{2}\beta_{2}\eta_{2}).$

Our model predicts that regime shifts in phage-microbe ecosystems can be a consequence of differences in species’ yields *Y*_2_ > *Y*_1_ rather than their burst sizes. A negative correlation between species’ growth rate and its yield known as rate-yield trade-off is widely known (15). According to this correlation, slower growing species tend to have higher yields, thereby facilitating bistability in our model.

A general case of predator-prey food webs with multiple trophic levels has been considered in references 16 and 17. For certain combinations of parameters, one can prove that the steady state of dynamical equations describing such ecosystems is unique and thus multistability is impossible. This proof, based on the Lyapunov function proposed in reference 18, requires the food web to have identical stoichiometry products (like *Y_i_*β*_i_* in our model) for all paths connecting the same pair of species. Here we extend this study by showing that if the difference in stoichiometries of two such paths is sufficiently large, multistability could in principle emerge. Thus, it is tempting to extend our mechanism for multistability up from microscopic phage-bacterium ecosystems to macroscopic predator-prey food webs. In order for macroscopic food webs to be multistable, the biomass conversion ratio between two successive trophic levels has to deviate widely from its typical value of about 10% (19, 20) and be sufficiently different for different species in the same trophic level. Indeed, one could always choose to measure the population of each species in units of its biomass per unit area. These units would rescale absolute values of competition parameters such as λ and η. In these units, stoichiometric coefficients *Y* and β are given by the efficiency (0% to 100%) of biomass conversion between two consecutive trophic levels. Multistability requires sufficient differences in biomass conversion factors along paths between species in different trophic levels. For example, in our model, the nutrient, which can be thought to occupy trophic level 0 is connected to the phage species (trophic level 2) via paths going through two different bacterial species (intermediate trophic level 1). Furthermore, the number of species in intermediate trophic levels of these paths has to be an odd number. Given that the overall number of trophic levels rarely exceeds 4, the case of a single intermediate trophic level considered in this study represents the most biologically plausible scenario.

**FIG 4:**
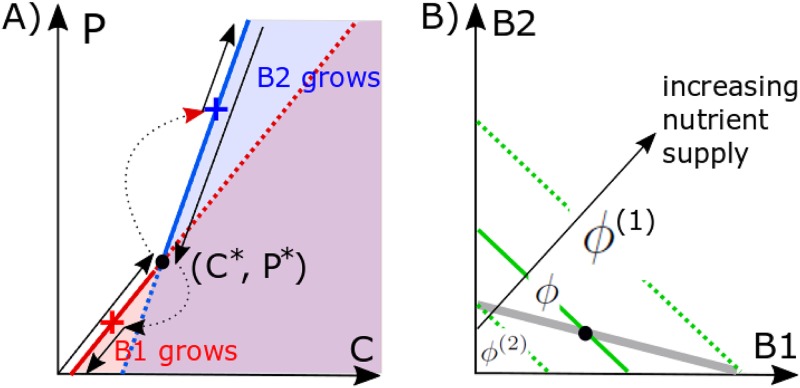
Geometric solution of the steady state of the ecosystem. (A) Steady-state *C* and *P* are from solving equations 2 and 3. When both bacteria *B*_1_ and *B*_2_ are present, the system can only be at the intersection (C*, P*). In our case, this state is dynamically unstable. As *ϕ* increases, the environmental parameters (*C*, *P*) follow the solid red line up to the black circle, then discontinuously jump to the blue cross, and continue up along the solid blue line. When *ϕ* subsequently decreases in the hysteresis loop shown in Fig. 2, the (*C*, *P*) follow the solid blue line down to the black circle, discontinuously jump to the red cross, and continue down along the solid red line. This trajectory is shown in black lines with arrows. (B) The geometric solution for coexisting bacterial populations is given by the intersection of the gray line, where the phage population is at the steady state *P* = P* (equation 14), and the green line, where the nutrient concentration is at the steady-state *C* = C* (equation 15). The green line shifts up as the nutrient supply ϕ is increased. Bacterial populations *B*_1_ (*B*_2_) disappear at the boundaries ϕ^(1)^ (ϕ^(2)^) of the bistability region ϕ^(2)^ < ϕ < ϕ^(1)^. Here we show an example in which the steady-state C*, P* is dynamically unstable, giving rise to bistability. However, if the gray line has a steeper slope than the green line, the bistability is replaced by the region (ϕ^(1)^ < ϕ < ϕ^(2)^) of stable coexistence of *B*_1_ and *B*_2_.

The ecosystem used in our study is very simple: it has low species diversity and a single growth-limiting nutrient. This simplicity allowed us to quantitatively understand the principal mechanisms giving rise to bistability. More-complex ecosystems with a larger number of species and multiple nutrients are expected to have qualitatively similar properties. They also could have a much more complicated phase diagram in the space of nutrient supply rates. Hence, multistability with more than two stable states could be realized in some regions of this space (see reference 4 for this type of multistability in consumer resource models). Another limitation of our model is that it ignores the possibility of rapid evolution of bacterial strains competing with phages. Such red queen dynamics often generates phage-resistant bacterial strains. The appearance of a phage-resistant variant of *B*_1_ would modify the behavior of our ecosystem for very high nutrient supply, but it might not affect bistability between *B*_1_ and *B*_2_ for intermediate nutrient supply studied above. This depends on the magnitude of the growth deficiency of the resistant mutant. A delicate interplay between multiple strains and species could be understood by visualizing them all in Fig. 4A, where a phage-resistant strain would be shown as a vertical line.

It is instructive to compare the mechanisms of bistability in our model to two previously described bistable systems involving phages and bacteria. One example of alternative stable states in a phage-microbe ecosystem has been described in reference 21. Unlike in our model, where regime shifts change the composition of bacterial species, the ecosystem modeled in reference 21 switches between the states with and without phages. The main feature responsible for this switching behavior is a decrease of adsorption coefficient of the bacterial host when nutrients become scarce. Similar to regime shifts in our ecosystem, the feedback between the nutrient concentration and the abundance of phages is at the core of this bistable behavior.

Perhaps the most celebrated example of a bistable system is the genetic switch operating inside a bacterial host of a temperate phage (22, 23). In a host of the prophage λ, there is an intracellular competition between the dormant, lysogenic state dominated by the repressor protein C1 (24) and the virulent, lytic state dominated by the protein Cro (25). When Cro wins, it leads to production of a large number of phages, akin to species *B*_2_ in our microbial ecosystem. High nutrient concentration in the environment typically favors the lytic state of the λ host (26). Such lytic state is analogous to the *B*_2_-dominated regime in our ecosystem, also favored by high *C*. In this sense, our ecosystem can be in the “dormant state,” producing few phages when it is dominated by *B*_1_. When this state is exposed to a strong pulse of *P*, *C*, or *B*_2_ it can switch to the “lytic state” dominated by *B*_2_ and producing many phages (Fig. 3).

One realistic implementation of bistability predicted by our model is in a phage-microbe ecosystem consisting of a bacterial strain protected against phages by the abortive infection (Abi) mechanism (*B*_1_) and a partially resistant strain (*B*_2_) coinfected by the same phage. Hosts with abortive infection allow phages to enter and kill them without producing a noticeable phage progeny (13). An example of the Abi defense is provided by certain types of CRISPR defense (27–29), where phages kill most of infected hosts but have zero or small burst size. In contrast to Abi- or CRISPR-protected bacteria, partially resistant strains may arise due to a mutation in the receptor protein which reduces both the growth rate (30) and the phage adsorption but has little effect on the burst size. Thus, regime shifts may naturally occur as a consequence of diverse phage defense mechanisms in microbial ecosystems (31).

A potential application of our system is in a new type of phage therapy in which phages targeting the pathogenic species (*B*_1_) are introduced together with carefully selected nonpathogenic species (*B*_2_) infected by the same phage. This therapy effectively combining two population pulses shown in Fig. 3A and C would lead to a more efficient and permanent elimination of the fast-growing pathogen (*B*_1_). One of the advantages of this approach is that phages would be continually present in the former patient, thereby preventing reentry of pathogenic bacteria. The strategy could be made even more favorable if the bacteria added together with phages could use a nutrient other than *C*, rendering it not vulnerable to nutrient competition from the pathogen.

## MATERIALS AND METHODS

### Simulations.

This paper investigates the dynamics of a model defined by equations 1 to 4, built on assumptions of mass action kinetics in a well-mixed system with an adjustable nutrient supply rate (32). We performed both deterministic and stochastic simulations of this model.

In stochastic simulations shown in Fig. 1B, we use the Gillespie algorithm with a step size of 0.0002 and rates defined for each of the nine basic processes in equations 1 to 4: nutrient introduction and dilution events, *B*_1_ and *B*_2_ replication events, phage infection events separately in *B*_1_ and in *B*_2_, and combined death/decay/dilution events in each of the two bacterial species and one phage species. Notice that a single phage infection event reduces the bacterial population by the step size equal to 0.0002 but increases the phage population by 0.0002β. A large value of the burst size *β*_2_ = 40 justifies a small step size used in our simulations.

Deterministic simulations shown in Fig. 2 solve the dynamics given by equations 1 to 4. At each value of nutrient supply rate ϕ, we integrate the equations for 1,000 time units to eliminate transients. We then increase the nutrient supply rate in increments *Δϕ* = 0.01. We use the steady-state populations/concentrations obtained at ϕ as starting populations/concentrations for simulations at *ϕ* + *Δϕ*.

Each blue or red circle in Fig. 3 was obtained by starting the system in one of the stable states, and subsequently changing one of the variables (*P*, *C*, *B*_1_ or *B*_2_) as indicated on the *y* axis. After a deterministic simulation of dynamic equations 1 to 4, for 1,000 time units, the final state is compared to each of the states possible for a given value of ϕ and is marked with the corresponding color in Fig. 3.

### Conditions for bistability.

In our model, it is convenient to describe the growth of a microbial species in (*C*, *P*) coordinates, characterizing the nutrient and phage concentrations in the environment, respectively. The population of a species grows exponentially for $\lambda C-\eta P>\delta_{B}$, decays exponentially for $\lambda C-\eta P<\delta_{B}$, and stays constant for $\lambda C-\eta P=\delta_{B}$. The last equation defines the so-called zero net growth isocline (ZNGI) (14) of the species defined by all environmental parameters where the population of this species could be in a steady state. Everywhere in the region of the (*C*, *P*) plane located to the right and below of species ZNGI (high *C* and small *P*), its population grows exponentially, while in the region to the left and above its ZNGI (low *C* and large *P*), it decays exponentially.

The red and blue straight lines in Fig. 4A correspond to the zero net growth isoclines of the fast- and the slow-growing bacterial species in our model, respectively. They intersect at the point (C*,P*) given by

(8) $$C^{*}=\frac{\delta_{B}(\eta_{1}-\eta_{2})}{\lambda_{1}\lambda_{2}\left(\frac{\eta_{1}}{\lambda_{1}}-\frac{\eta_{2}}{\lambda_{2}}\right)}$$

(9) $$P^{*}=\frac{\delta_{B}(\lambda_{1}-\lambda_{2})}{\lambda_{1}\lambda_{2}\left(\frac{\eta_{1}}{\lambda_{1}}-\frac{\eta_{2}}{\lambda_{2}}\right)}$$

The intersection point corresponds to the only set of environmental parameters at which these two species can potentially coexist with each other.

The lower part of species 1 ZNGI (the solid part of the red line) extending from *P* = 0 and up to the intersection point at P* and the upper part of species 2 ZNGI above P* (the solid part of the blue line) have a special property that the other species would not be able to grow in this environment. Hence, the union of these two halves of ZNGIs corresponds to uninvadable states of the ecosystem, which are the main focus of this study. The exact position of the environmental parameters on the (*C*, *P*) plane is determined by the supply rate *ϕ* of the limiting nutrient to the ecosystem. For $\phi <\delta_{C}\delta_{B}/\lambda_{1}$, there is not enough nutrient to support the growth of any species, and the environment remains abiotic. Hence, the first transition happens at

(10) $$\phi_{B1}=\frac{\delta_{C}\delta_{B}}{\lambda_{1}}$$

For $\phi_{B1}<\phi <\delta_{C}\delta_{B}/\lambda_{1}+\delta_{P}\delta_{B}/(Y_{1}\beta_{1}\eta_{1}),$ species 1 is present, but its biomass is not sufficient to support the survival of the phage. The phage first enters the ecosystem at

(11) $$\phi_{P1}=\frac{\delta_{C}\delta_{B}}{\lambda_{1}}\left(1+\left(\frac{\delta_{P}}{\delta_{C}}\right)\left(\frac{\lambda_{1}}{Y_{1}\beta_{1}\eta_{1}}\right)\right)$$

For even larger nutrient supply rates, $\phi_{P1}<\phi <\phi^{\left(1\right)}=C^{*}\delta_{P}\left(\frac{\lambda_{1}}{Y_{1}\beta_{1}\eta_{1}}+\frac{\delta_{C}}{\delta_{P}}\right),$ the ecosystem contains only species 1 and the phage. The crucial parameters of the phage-bacterium ecosystem considered in our model are

(12) $$\phi^{\left(1\right)}=C^{*}\delta_{P}\left(\frac{\lambda_{1}}{Y_{1}\beta_{1}\eta_{1}}+\frac{\delta_{C}}{\delta_{P}}\right)$$

(13) $$\phi^{\left(2\right)}=C^{*}\delta_{P}\left(\frac{\lambda_{2}}{Y_{2}\beta_{2}\eta_{2}}+\frac{\delta_{C}}{\delta_{P}}\right)\;$$

where C* is given by equation 8. For nutrient supply rates *ϕ* >*ϕ*^(1)^, species 2 can in principle grow in the ecosystem given *C* and *P* shaped by species 1. What happens in this region crucially depends on whether or *ϕ*^(1)^ >*ϕ*^(2)^, with the latter case corresponding to bistability, which is the main focus of this study. For pedagogical reasons, let us first consider the model where *ϕ*^(1)^ <*ϕ*^(2)^ and thus *B*_1_-*B*_2_ coexistence is possible. In this case, both species 1 and 2 can coexist with each other in the interval $C^{*}\delta_{P}\left(\frac{\lambda_{1}}{Y_{1}\beta_{1}\eta_{1}}+\frac{\delta_{C}}{\delta_{P}}\right)=\phi^{\left(1\right)}<\phi <\phi^{\left(2\right)}=C^{*}\delta_{P}\left(\frac{\lambda_{2}}{Y_{2}\beta_{2}\eta_{2}}+\frac{\delta_{C}}{\delta_{P}}\right).$ The abundances of each of the two microbial species can be geometrically determined as the intersection of two straight lines in the (*B*_1_, *B*_2_) plane shown in Fig. 4B The gray line corresponds to the steady state of the phage population *P* in equation 4 and is given by the equation

(14) $$\beta_{1}\eta_{1}B_{1}+\beta_{2}\eta_{2}B_{2}=\delta_{P}\;$$

It must intersect with another straight line defining the steady state of the nutrient concentration *C *= C* and is given by

(15) $$\frac{\lambda_{1}B_{1}}{Y_{1}}+\frac{\lambda_{2}B_{2}}{Y_{2}}=\frac{\phi }{C^{*}}-\delta_{C}\;$$

These lines intersect for positive *B*_1_ and *B*_2_ when *ϕ*^(1)^ < *ϕ*< *ϕ*^(2)^.

In the opposite case, where *ϕ*^(1)^ >*ϕ*^(2)^, the system is capable of bistability for nutrient supply rates *ϕ*^(2)^ <*ϕ* <*ϕ*^(1)^. To understand this, it is useful to follow the trajectory of environmental parameters (*C*, *P*) as *ϕ* is gradually increased. For $\phi_{P1}<\phi <\phi^{\left(1\right)},$ the environmental parameters follow the ZNGI of the fast-growing species 1 (the red line in Fig. 4A below the intersection with the blue line). Immediately above the intersection point (C*, P*), realized for nutrient supply rate slightly larger than *ϕ*^(1)^, the ecosystem becomes invadable by species 2. However, for this species, the intersection point (C*, P*) corresponds to a lower value of nutrient supply *ϕ*^(2)^ < *ϕ*^(1)^. Hence, after a brief transient period, the environmental parameters (*C*, *P*) of our ecosystems move to the position marked with the blue cross in Fig. 4A. As *ϕ* continues to increase above *ϕ*^(1)^, the environmental parameters follow the ZNGI of species 2 (the blue line to the right of the blue cross in Fig. 4A).

If at some point one starts decreasing *ϕ*, species 2 will persist down to *ϕ*^(2)^ at which the environmental parameters are again at the coexistence point (C*, P*). For slightly lower ϕ, the environmental parameters will discontinuously jump to the point marked with the red cross on the ZNGI of species 1. For even lower nutrient supply rates, they will continue to follow the ZNGI of species 1 to the left and below the red cross. Hence, our environment is bistable in the interval of two ZNGIs between the red and blue crosses. The lower red part of this interval is reachable only when *ϕ* is increased from a low value below *ϕ*^(2)^, while the upper blue part is reachable only when *ϕ* is decreased from a high value above *ϕ*^(1)^.

Above we assumed that phages can survive for *ϕ* = *ϕ*^(2)^ in the ecosystem dominated by species 1 instead of species 2. This requires $C^{*}\delta_{P}\left(\frac{\lambda_{2}}{Y_{2}\beta_{2}\eta_{2}}+\frac{\delta_{C}}{\delta_{P}}\right)=\phi^{\left(2\right)}>\phi_{P1}=\frac{\delta_{C}\delta_{B}}{\lambda_{1}}\left(1+\left(\frac{\delta_{P}}{\delta_{C}}\right)\left(\frac{\lambda_{1}}{Y_{1}\beta_{1}\eta_{1}}\right)\right)$, which can be rewritten as

(16) $$\frac{\eta_{1}-\eta_{2}}{\frac{\lambda_{2}}{\lambda_{1}}\eta_{1}-\eta_{2}}>\frac{\frac{\lambda_{1}}{Y_{1}\beta_{1}\eta_{1}}+\frac{\delta_{C}}{\delta_{P}}}{\frac{\lambda_{2}}{Y_{2}\beta_{2}\eta_{2}}+\frac{\delta_{C}}{\delta_{P}}}\;$$

In the opposite limit of this inequality and for nutrient supply rates satisfying $\phi^{\left(2\right)}<\phi <\phi_{P1},$ the phages will be absent in one of the two alternative stable states (dominated by species 1) but present in another one (dominated by species 2).

The scenario illustrated in Fig. 2 corresponds to $\phi_{P1}<\phi^{\left(2\right)}<\phi^{\left(1\right)}.$ In this case, the abundances in the steady-state *S* dominated by the fast-growing species 1 are given by

(17) $$B_{1}^{\left(F\right)}=\frac{\delta }{\beta_{1}\eta_{1}}$$

(18) $$B_{2}^{\left(F\right)}=0$$

(19) $$C^{\left(F\right)}=\frac{\phi }{\delta +\lambda_{1}B_{1}^{\left(F\right)}/Y_{1}}$$

(20) $$P^{\left(F\right)}=\frac{\lambda_{1}C^{\left(F\right)}-\delta }{\eta_{1}}$$

The abundances in the alternative stable state *F* dominated by the slow-growing species 2

(21) $$B_{1}^{\left(S\right)}=0$$

(22) $$B_{2}^{\left(S\right)}=\frac{\delta }{\beta_{1}\eta_{1}}$$

(23) $$C^{\left(S\right)}=\frac{\phi }{\delta +\lambda_{2}B_{2}^{\left(S\right)}/Y_{2}}$$

(24) $$P^{\left(S\right)}=\frac{\lambda_{2}C^{\left(S\right)}-\delta }{\eta_{2}}$$

in the state dominated by species 2.

In the regime where $\phi_{P1}<\phi^{\left(2\right)}<\phi^{\left(1\right)}$ and for nutrient supply rates in the bistable window *ϕ*^(2)^ <*ϕ*<*ϕ*^(1)^, the ecosystem also has a dynamically unstable steady state in which both bacterial species coexists with each other and have the following abundances:

(25) $$B_{1}^{\left(U\right)}=\left(\frac{\delta }{\beta_{1}\eta_{1}}\right)\left(\frac{\phi -\phi^{\left(2\right)}}{\phi^{\left(1\right)}-\phi^{\left(2\right)}}\right)$$

(26) $$B_{2}^{\left(U\right)}=\left(\frac{\delta }{\beta_{2}\eta_{2}}\right)\left(\frac{\phi^{\left(1\right)}-\phi }{\phi^{\left(1\right)}-\phi^{\left(2\right)}}\right)$$

Note that in our study we consider only uninvadable states of the ecosystem. In other words, we ignore an invadable steady state, where for a small value of *ϕ*, the ecosystem is populated only by species 2, or another invadable steady state realized for a large value of *ϕ*, where the ecosystem has only species 1. These states are located on invadable parts of each species’ ZNGI, which are to the right and below the ZNGI of the other species in Fig. 4A. Indeed, in these regions an arbitrary small inoculum of the invading species would exponentially grow and thereby disrupt the steady state of the ecosystem, moving the environmental variables to a new point on the (*C*, *P*) plane.

### Parameters used in our numerical simulations.

Both in stochastic and deterministic simulations of our model shown in Fig. 1 to 3, we used the following parameters:

(27) $$\lambda_{1}=1.0\;\text{and}\;\lambda_{2}=0.8$$

(28) $$Y_{1}=1\;\text{and}\;Y_{2}=1$$

(29) $$\eta_{1}=0.2\;\text{and}\;\eta_{2}=0.15$$

(30) $$\beta_{1}=2\;\text{and}\;\beta_{2}=40$$

(31) $$\delta_{C}=\delta_{B}=\delta_{P}=0.2$$

For these parameters, the ecosystem is bistable when the nutrient supply rate is between

(32) $$\phi^{\left(2\right)}=0.2266$$

(33) $$\phi^{\left(1\right)}=0.7$$

The bacterial abundances anywhere within this interval of nutrient supply rates are given by $B_{1}^{\left(F\right)}=0.5\;\text{and}\;B_{2}^{\left(F\right)}=0$ or $B_{1}^{\left(S\right)}=0\;\text{and}\;B_{2}^{\left(S\right)}=0.0333$ in alternative stable states dominated by the fast-growing and the slow-growing bacterial species, respectively.

The other transitions visible in Fig. 2 happen at *ϕ_B_*_1_ = 0.04 above which bacterial species 1 is able to survive given the dilution rate *δ*, and *ϕ_P_*_1_ = 0.14, above which the phage can survive in this ecosystem.

To estimate the typical values of *C* and *P* in two bistable states, let us consider one example when *ϕ* = 0.25 is slightly above *ϕ*_(2)_. In this case, the steady-state concentrations of the nutrient and the phage in two alternative stable states: *F* and *S* are given by

(34) $$C^{\left(F\right)}=0.357\;\text{and}\;C^{\left(S\right)}=1.277$$

(35) $$P^{\left(F\right)}=0.786\;\text{and}\;P^{\left(S\right)}=5.476$$

The dynamically unstable steady-state point always has C* = 1 and P* = 4, which are located between their values in the *F* and *S* states. The bacterial abundances in an unstable state for *ϕ* = 0.25 are given by $B_{1}^{\left(U\right)}=0.0246$ and $B_{2}^{\left(U\right)}=0.0317$. Note that the steady-state abundance of species 1 in the unstable state is much lower than its abundance $B_{2}^{\left(U\right)}=0.0317$ in the stable state. That suggests why for such a low value of *ϕ* we found it impossible to switch the ecosystem from the *F* state to the *S* state by pulses of *C*, *P*, or *B*_2_. Indeed, neither of these transient pulses is capable of lowering *B*_1_ down to the extra low saddle point value $B_{2}^{\left(U\right)}=0.0317$ from the initial stable state value of $B_{1}^{\left(F\right)}=0.5$ without simultaneously moving the populations of other species away from the saddle point region.

The positions of the crosses in Fig. 4A can be calculated as follows: at ϕ = ϕ^(1)^ = 0.7, species 1 sets the environmental parameters of the ecosystem exactly at the intersection point (C*,P*) = (1,4) between ZNGIs of species 1 and 2. For slightly higher nutrient supply rates, species 2 eliminates species 1 and the nutrient concentration shifts to $C_{2x}=\frac{\phi^{\left(1\right)}}{\delta_{C}+\delta_{P}\lambda_{2}/(Y_{2}\beta_{2}\eta_{2})}=3.09$, and the phage population shifts to $P_{2x}=(\lambda_{2}C_{2x}-\delta_{B})/\eta_{2}=15.14$. On the way down, bacterial species 1 reenters the ecosystem slightly below *ϕ* = *ϕ*^(2 )^ = 0.2266. When species 1 replaces species 2 immediately below this point, the nutrient concentration shifts to $C_{1x}=\frac{\phi^{\left(2\right)}}{\delta_{C}+\delta_{P}\lambda_{1}/(Y_{1}\beta_{1}\eta_{1})}=0.3238$, and the phage population shifts to $P_{1x}=(\lambda_{1}C_{1x}-\delta_{B})/\eta_{1}=0.6190$.
